# The Feasibility of Immunocryosurgery in the Treatment of Non-Superficial, Facial Basal Cell Carcinoma That Relapsed after Standard Surgical Excision: An Experience Report from Two Centers

**DOI:** 10.3390/curroncol29110668

**Published:** 2022-11-07

**Authors:** Georgios Gaitanis, Athanasia Zampeta, Panagiota Tsintzou, Grigorios Fillis, Konstantinos Seretis, Laurence Feldmeyer, Ioannis Bassukas

**Affiliations:** 1 Department of Skin and Venereal Diseases, Faculty of Medicine, School of Health Sciences, University of Ioannina, 45100 Ioannina, Greece; 2 DELC Clinic, 2502 Biel/Bienne, Switzerland; 3 Department of Plastic and Reconstructive Surgery, Faculty of Medicine, School of Health Sciences, University of Ioannina, 45100 Ioannina, Greece; 4 Department of Dermatology, Inselspital, Bern University Hospital, University of Bern, 3010 Bern, Switzerland

**Keywords:** immunocryosurgery, imiquimod, cryosurgery, basal cell carcinoma

## Abstract

In this retrospective, chart review study, we evaluated the feasibility of immunocryosurgery in facial, non-superficial basal cell carcinomas (BCC) that had relapsed after standard surgery. Inclusion criteria were (a) ‘biopsy confirmed relapse of facial BCC’, (b) known ‘calendar year of surgical excision(s)’, and (c) ‘relapse within 10 years after the last surgical excision’. Tumors treated from 1 January 2011 to 31 December 2020 with a standard 5-week immunocryosurgery cycle (daily imiquimod application for 5 weeks and a cryosurgery session at day 14) were included. Descriptive statistics, Kaplan–Meier method, and Cox proportional hazards model were calculated with significance at *p* < 0.05. From the *n* = 27 BCC evaluated, *n* = 20 (74.1 ± 8.4%) cleared after one immunocryosurgery cycle. Two of the remaining cases cleared completely after a repeat cycle, one patient favored surgery, and four BCC did not clear despite additional immunocryosurgery cycles (feasibility 81.5 ± 7.5%). Of the 22 tumors with clinical outcome ‘complete clearance with immunocryosurgery’, three BCC relapsed at 9, 28, and 50 months. Overall, the 5–year treatment efficacy rate was 60.2 ± 13.4% (mean follow-up 94.6 ± 15.1 months). In total, 20/27 BCC relapses after surgery (74.1%) were tumor-free at the end of personalized follow-up times (66.7 ± 12.4% tumor free patients at 5-year follow-up). Number of tumor relapses before immunocryosurgery was the single predictor of tumor progression after immunocryosurgery (*p* = 0.012). Conclusively, immunocryosurgery could be further evaluated as an alternative, definitive treatment of selected facial BCC relapsing after surgery.

## 1. Introduction

Ιmmunocryosurgery is a minimally invasive therapeutic approach that utilizes the synergistic potential of a fixed timing combination of cryosurgery and topical imiquimod. A standard immunocryosurgery treatment cycle consists of once-daily 5% imiquimod cream application for 5 weeks and a rather mild cryosurgery session at day 14 of the imiquimod course [1]. Immunocryosurgery has demonstrated efficacy in the treatment of primary, non-superficial, basal cell carcinomas (BCC) with cure rates comparable to that of standard surgery [1,2,3,4]. Thus, after only one treatment cycle with immunocryosurgery, the 5-year recurrence rate of primary BCC with a maximal diameter ≤2 cm was 91.4 ± 2.8% for the per protocol and 87.7 ± 3.1% for the per intention to treat analyses [3]. Notably, with immunocryosurgery repeat cycles for partial responders and relapses, only three BCC sites were not tumor free at last follow-up (effectiveness: 97.1 ± 1.6% per protocol or 93.2 ± 2.3% per intention to treat analyses) [3]. Moreover, neither tumor size (tumor diameter smaller vs. larger than 1.0 cm; *p* = 0.865) nor tumor localization (within vs. outside the H-region of the face; *p* = 0.233) seemed to modulate the treatment outcome [2,3]. The distinct efficacy of immunocryosurgery can most probably be attributed to the particular timing of the combination [5] as ‘cryosurgery during imiquimod application’ proved to be more effective compared to other timing protocols (e.g., ‘cryosurgery preceding imiquimod application’) [6]. At the tissue level, the cryoablation of the preconditioned from imiquimod application tumor seemed to abrogate the expected immune tolerance phenomena that continued imiquimod application induces and triggered a vivid specific antineoplastic inflammatory response [7]. The latter is both temporally and spatially mostly confined to the treated tumor. Corresponding alterations in the profile of cytokines and immune cells in the blood reflect this induction of immunoenhancing effects during the local treatment of BCC [8]. Due to its satisfactory feasibility including the treatment of older and frail patients and the excellent safety profile, immunocryosurgery is gaining recognition and is used in the outpatient setting in addition to the treatment of BCC and Bowen’s disease in immunocompetent and immunocompromised patients [9,10,11] as well as in the therapy of selected cases of *lentigo maligna* [12,13], Merkel cell carcinoma [14], and squamous cell carcinoma [15]. Of note, immunocryosurgery is a tissue sparing modality whose efficacy is not really limited by the tumors’ localization, tumor histology, or patients’ overall health condition [1,16].

BCC relapses after surgery, standard excision or Mohs’ procedure, constitute a subset of challenging, difficult to treat tumor cases, for which the standard of treatment recommendation is Mohs’ micrographic surgery [17]. However, Mohs’ surgery [17,18] is resource-intensive, not widely available and, in addition, there are recently rising concerns that it may carry an increased risk for the possible overtreatment of elderly BCC patients with limited life expectancy [19].

In the past decade, we had sporadically treated relapsed BCC with immunocryosurgery (including five already reported cases [20,21]) with promising efficacy and adequate tissue sparing results. In the present retrospective study, based on the compilation of these latter cases, we sought to evaluate the feasibility of immunocryosurgery as performed in a tertiary hospital (Ioannina, Greece) and an ambulatory clinic (Clinic DELC, Biel/Bienne, Switzerland) to treat BCC relapsing after standard surgical excision.

## 2. Materials and Methods

The University Hospital of Ioannina Institutional Review Board Committee granted permission (Approval No. 6/17.02.2022, θ.26 from 17 February 2022) and the files of patients with relapsed BCC treated with immunocryosurgery from 1 January 2011 to 31 December 2020 in the Dermatology Department of the Hospital as well as in an outpatient dermatology center in Switzerland (Dermatologie EsthétiqueLaser Chirurgie, [DELC Clinique] Biel/Bienne) were included in the study. All patients prior to treatment with immunocryosurgery were offered as a first choice the repeat of standard surgical excision. Notably, immunocryosurgery was introduced in the latter of the above centers in 2019, so in addition to restricted patient numbers, the relevant follow-up time was not expected to be more than 24 months. Inclusion criteria were (a) ‘relapse of a non-superficial, facial BCC’ (i.e., biopsy proven BCC tissues within or in contact with the surgical scar of a previously surgically treated and biopsy confirmed facial BCC), (b) known ‘calendar year of surgical excision(s) in the past’, and (c) ‘relapse within a 10-year period after the last surgical excision’. This latter criterion was specifically included to avoid the inclusion of new tumors that could have possibly developed in the direct proximity of the postsurgical scars and thus would constitute a new BCC and not a relapse. The results as per 31 December 2021 are reported.

A non-ablative therapeutic modality as an alternative to surgical treatment for BCC must present with an adequately high rate of complete tumor clearance (‘cure’) [22]. Accordingly, we differentiated between two distinct treatment failure endpoints for the evaluation of the overall feasibility of immunocryosurgery in the present setting: (a) ‘no achievement of complete tumor clearance’ (no ‘complete response’, no CR), and (b) ‘relapse’ of a ‘complete responder’ during subsequent follow-up. Presently, for the *per intension* to treat analysis of the efficacy of immunocryosurgery, the above disease-progression events were analyzed both separately and jointly. After treatment, the tumor sites were followed up clinically for relapses including dermoscopy and optical coherence tomography in some cases. Relapses of BCC after immunocryosurgery appear as an enlarging papule within the scar of the previous treatment and present all the typical clinical and dermoscopic features of BCC. Therefore, follow-up was performed as in our previous studies [2,3,6], with clinical and dermatoscopic examination of the treated sites and in a case of a suspected recurrence, a biopsy was performed. Times to first disease progression event or to last follow-up information were calculated from the moment of the initiation of the first immunocryosurgery treatment cycle. For non-complete responders, the times-to-event were arbitrarily set at 0 (i.e., the moment of the initiation of the immunocryosurgery treatment). However, evaluation of a no-CR can be conducted at the 3-month follow-up appointment, in which immunocryosurgery-induced inflammation has subsided and tumor remnants are detectable during clinical examination including dermoscopy of the area.

In all cases, treatment started with a standard 5–week immunocryosurgery cycle that involved the daily application of imiquimod for 5 weeks and a relatively mild cryosurgery session (liquid N_2_, open spray, two cycles of 15 s each) at day 14. The cryosurgery technique has recently been described in detail [1,11]. Incomplete responses and relapses after immunocryosurgery were scheduled to be treated with (repeat) immunocryosurgery cycles [11]; however, surgery or radiotherapy was also offered according to patient preferences. Once vismodegib was made available, it was also suggested as an alternative.

Data are summarized with descriptive statistics. Times-to-events were analyzed employing the Kaplan–Meier calculator and Cox proportional hazards model (backward stepwise Wald method) using SPSS (Statistics for Windows; IBMCorp., Armonk, NY, USA). Statistical significance was set up at *p* < 0.05.

## 3. Results

*n* = 30 BCC were identified in the databases, which were referred to as relapses after surgery and treated with immunocryosurgery. In the final analysis, *n* = 27 tumors were included (Table S1, Supplementary Materials); two cases were excluded due to unknown calendar year of reported surgery and in one case, the instigated surgical intervention was dated more than 10 years earlier (20 years). Twenty-three tumors were treated in the Department of Skin and Venereal Diseases in the University Hospital of Ioannina and the remaining four in DELC. From the 27 relapsing tumors, six BCC (22%) were of aggressive histology: four neoplasms were of basosquamous, one of mixed (basosquamous and micronodular), and one of keratotic micromorphology (Table S1).

**Figure 1:**
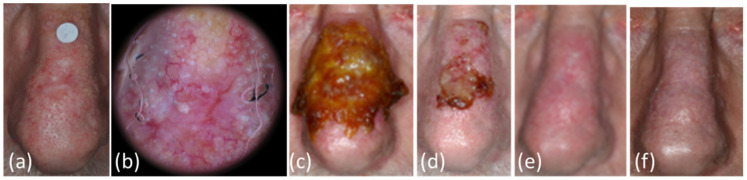
Patient/tumor 14 (Table S1, Supplementary Materials). (**a**,**b**) Clinical and dermoscopic picture of the relapsed basal cell carcinoma one year after surgery in a 67 year old patient. The tumor diameter was measured at 15 mm with the absence of clear borders. Solar damage of the surrounding skin of the nose was also evident. (**c**) After 2 weeks of daily imiquimod treatment at the day of cryosurgery treatment. There was extended skin inflammation due to the application of imiquimod on the whole of the nose. The cryosurgery was spatially confined on the initial tumor area and a narrow skin rim around it. (**d**) At the last day of imiquimod application (day 35), end of treatment. The intense inflammatory response had subsided. (**e**) At the one-month post-treatment follow-up appointment. (**f**) Thirty-six months after the end of treatment: no relapse and an excellent cosmetic outcome were recorded.

Twenty tumors (percent ± standard error: 74.1 ± 8.4%) cleared after one immunocryosurgery treatment cycle (exemplary case in Figure 1).

**Figure 2:**
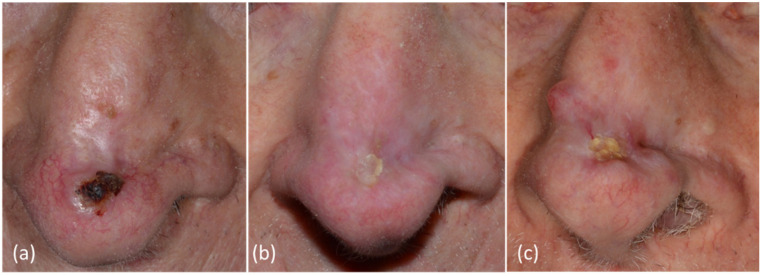
Patient/tumor 12 (Table S1, Supplementary Materials). (**a**) Basal cell carcinoma relapse after surgery in an 86–year-old patient at baseline. (**b**) Twelve months after initiation of immunocryosurgery treatment cycles: partial response; (**c**) At 44 months of follow-up: slow disease progression. At this point, the already 90–year-old patient denied any further therapeutic interventions.

From the remaining seven tumors, two cases cleared completely after a repeat immunocryosurgery cycle, one patient favored surgery at that point, and four BCC did not clear after additional immunocryosurgery treatment cycles (Figure 2) (i.e., the feasibility of immunocryosurgery to induce tumor clearance (cure) of relapsed BCC was 81.5 ± 7.5% (22/27 cases)).

**Figure 3:**
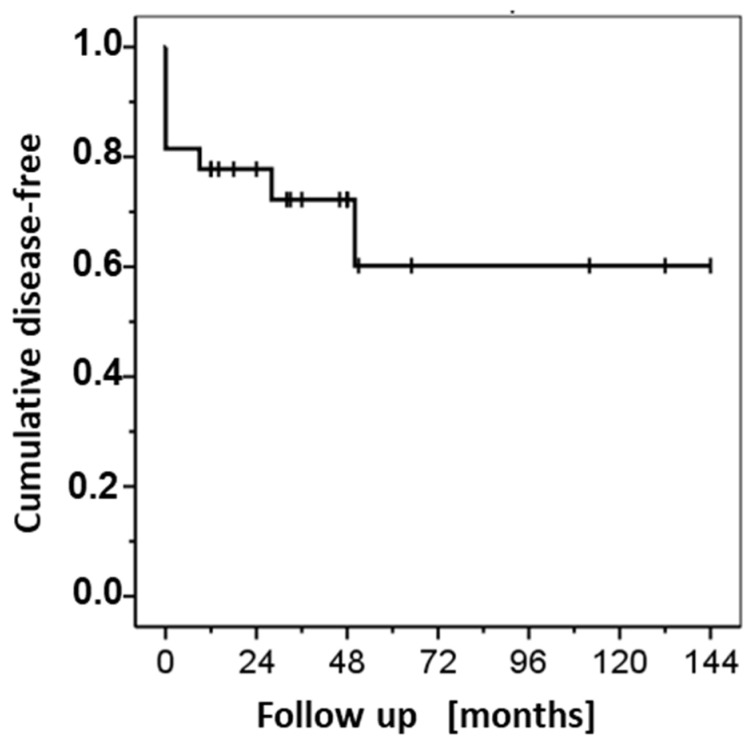
Immunocryosurgery for basal cell carcinoma relapses after surgery. Probability of disease-free tumor site as a function of follow-up time after the first immunocryosurgery treatment cycle (solid line). Short perpendicular bars represent points of censoring events (i.e., times to last available follow-up information of no relapse).

Of the four above patients with treatment outcome ‘no clearance’, one patient preferred surgery after the second immunocryosurgery treatment cycle. In the remaining three patients, stabilization of the achieved partial remission was accomplished with periodically repeated immunocryosurgery treatment cycles to the end of follow-up (two of them at 6 and 44 follow-up months) or to the time of vismodegib initiation (one patient at 67 months follow-up). Of the 22 tumors with clinical outcome ‘cure with immunocryosurgery’, three BCC relapsed at 9, 28, and 50 months, respectively, after treatment initiation. Taken together, the tumor relapses after immunocryosurgery and the never cleared cases with this modality correspond to an overall 5–year treatment efficacy rate of 60.2 ± 13.4% with a mean follow-up time without a disease progression event of 94.6 ± 15.1 months (Table 1 and Figure 3). Of the three tumor relapses after immunocryosurgery, one BCC cleared after a single immunocryosurgery treatment cycle repetition and two were referred for surgical treatment. In total, 20/27 BCC relapses after surgery (74.1%) were tumor-free at the end of personalized follow-up times. This corresponded to a 5-year disease free rate of 66.7 ± 12.4% after treatment exclusively with immunocryosurgery (respective mean time in sustained tumor control: 101.9 ± 14.2 months; Table 1 and Figure 3).

Stable disease at the end of the available follow-up time is an optional treatment outcome in the cohort of, in their majority, elderly patients with relapsed BCC. The overall efficacy of the proposed immunocryosurgery-based therapeutic approach to ensure at least stable disease to the end of the individual follow-up time was 85.2% (22/27 BCC). Only in 5/27 cases was this therapy revised in favor of other modalities (surgery in four cases and vismodegib in one).

The impact of potential predictors of immunocryosurgery effectiveness as a treatment modality for BCC relapses after standard surgical excision was evaluated with the Cox proportional hazards model. From the inserted regressors (age of the patients, maximal tumor diameter, average tumor growth rate, i.e., maximal tumor diameter/time after the last surgical treatment in mm/year, number of tumor relapses before immunocryosurgery and counts of risk factors for relapse before surgery) only the number of tumor relapses before immunocryosurgery was a statistically significant predictor of a tumor progression event (either no complete response or relapse) after starting immunocryosurgery (*p* = 0.012; Table S2, Supplementary Materials). Notably, treatment effectiveness did not differ between older (≥75 years old) and younger patients (*p* = 0.967, log rank test; Figure S1, Supplementary Materials).

The adverse events of the treatment included the ones anticipated during immunocryosurgery (inflammation at the treated site, oozing, and crusting, particularly during the time following the cryosurgery session) [1]. One patient complained about a limited flu-like episode without fever the day following the cryosurgery session.

## 4. Discussion

In the present retrospective study, two thirds (66.7%) of BCC that had relapsed after surgery were relapse-free with immunocryosurgery after 5 years of follow-up, a noticeable long-term treatment efficacy for tumors of this challenging cohort. Moreover, at least stable disease (mostly complete responses) was achieved in a remarkably high percentage of the tumors treated (85.2%) and only in five out of the 27 cases was the therapy revised in favor of surgery (four cases) or vismodegib (one case). The treatment of BCC relapses after surgery remains a therapeutic challenge. Guidelines for the treatment of BCC including the European guidelines [23], recommend Mohs’ micrographic surgery or radiotherapy for this BCC subset. However, as herein confirmed for immunocryosurgery, the efficacy of all therapeutic modalities including the effectiveness of Mohs’ surgery [13] is lower in this setting compared to that of the primary tumors. Nevertheless, the optimization of treatment selection for BCC in frail patients or patients with limited life expectancy is a crucial issue that demands redefinition of the treatment targets in order to avoid possible overtreatment with the use of disproportionally aggressive and burdensome procedures [19]. Our present findings further underline the impact of immunocryosurgery, an office- and, even, house-compatible modality, as an effective alternative to highly specialized treatment methods. Likewise, many of the tumors treated herein fall into the not-clearly defined category of “locally advanced BCC”, which would be suitable for hedgehog inhibitor treatment [24]. Interestingly, facial localization and size ≥2 cm were criteria for the inclusion of BCC cases in this category of locally advanced tumors, in a recent real world clinical study that comprised 433 BCC [25]. Although, not directly comparable, immunocryosurgery seems to achieve higher cure rates compared to hedgehog inhibitors, but lower than the ones reported for Mohs’ surgery of relapsed BCC [25].

An intriguing finding in the present study is that the number of relapses before immunocryosurgery was a predictive factor for a tumor relapse after immunocryosurgery (Table S2, Supplementary Materials). In comparison, other parameters such as patient age or tumor size did not affect the treatment outcome in the present study, probably due to the limited number of cases. However, unpublished data from our center show that BCC size can be a predicting factor in tumors with maximal diameter >2 cm, as the efficacy of a standard 5-week immunocryosurgery cycle falls significantly in larger tumors with a maximal diameter between 2 to 4 cm. Thus, for larger BCC, individualized approaches are required [1]. This supports previous observations that within the population of BCC exist tumors with deviated biological behavior exist that affects treatment susceptibility. Likewise, the BCC histological subtype is a well-known determinant of the effectiveness of surgery [26] but does not seem to play a similarly significant role with immunocryosurgery [2]. Accordingly, tumors carrying resistance mutations to hedgehog inhibitors fail treatment with these agents [27]. Imiquimod monotherapy also seems to differentiate two subgroups of BCC according to therapeutic response: those that achieve a lasting complete remission and those that fail to achieve clearance [28]. As immune mediated phenomena underly imiquimod monotherapy [29,30] and the proposed immunocryosurgery, any alterations in the tumor or patient immune profile may impact the response to treatment. Regarding immunocryosurgery, this is eloquently presented by the differential response of individual BCC within the same patient [21] and the limited restricted response of immunosuppressed patients [11]. These findings point to the presence of, on one hand, universal predictive ‘tumor-specific’ factors for the response of BCC to treatment, common to all different therapeutic modalities, destructive or not, and on the other, of modality-specific predictive factors of treatment failure. Knowledge of these factors may guide us in the future to the selection of the most appropriate for a particular tumor treatment modality. However, focusing on the elaboration of such ‘inherent’ factors that mediate global treatment resistance, independent of the employed therapeutic modalities, may help to understand the peculiarly low rate of aggressive BCC cases. In this latter framework, our present results suggest that history of multiple relapses after surgery is also a predictor of treatment failure with immunocryosurgery.

The major restrictions of the present study are its retrospective design and the heterogeneity of the tumors included.

## 5. Conclusions

Immunocryosurgery, which is a minimally invasive and highly efficacious modality for primary BCC irrespective of the localization or the tumor histology, could be evaluated in the future as an adjuvant treatment of selected relapsing BCC.

## Figures and Tables

**Table 1:** Probability of disease-free tumor sites (standard error, S.E.) as a function of follow-up time after treatment initiation with immunocryosurgery for basal cell carcinoma (BCC) that had relapsed after standard surgical excision (Kaplan–Meier method).

Follow-up [months]	Cases in Follow-up (% of Initial Cases)	BCC Sites not Disease Free (in Interval)	Disease-Free [S.E.] %	
			All Cases (*n* = 27)	Cleared BCC (*n* = 22) ^#^
0 *	22 (81.5)	5	81.5 [7.5]	100
12	21 (77.8)	1 ^&,$^	77.8 [8.0]	95.5 [4.4]
18	16 (59.3)	0		
24	15 (55.6)	0		
36	11 (40.7)	1 ^&,†^	72.2 [9.2]	
48	9 (33.3)	0		
60	4 (14.8)	1 ^&,$^	60.2 [13.4]	81.8 [13.2]

* 0 months: arbitrary time for no complete tumor clearance with immunocryosurgery (‘not cleared’). ^&^ Relapse. ^$^ Treatment with surgery. ^†^ Complete response with an immunocryosurgery treatment cycle. ^#^ Complete responders with immunocryosurgery.

## Data Availability

The data are contained within the article or supplementary material.

## Supplementary Materials

The following supporting information can be downloaded at: https://www.mdpi.com/article/10.3390/curroncol29110668/s1, Figure S1: Immunocryosurgery for BCC relapses after surgery: Disease free tumor sites for patients older from 75 years vs. younger. p: log rank (Mantel-Cox test).; Table S1: Patient and tumor characteristics included in the study.; Table S2: Multivariate comparison of predictors of the treatment outcome of basal cell carcinoma relapses after surgery with immunocryosurgery. Cox proportional hazards model for the prediction of a disease-free tumor site (‘clearance’) at last follow-up.
