# Supplementary material for: The Feasibility of Immunocryosurgery in the Treatment of Non-Superficial, Facial Basal Cell Carcinoma That Relapsed after Standard Surgical Excision: An Experience Report from Two Centers
Source: Curr Oncol. 2022 Nov 7;29(11):8475–82. doi: 10.3390/curroncol29110668 (PMC9688977; doi:10.3390/curroncol29110668)
Supplement: Supplementary file 1 [file curroncol-29-00668-s001.zip › curroncol-1948523-supplementary.pdf]

Current Oncology

Supplementals

The Feasibility of Immunocryosurgery in the Treatment of Non-Superficial, Facial Basal Cell Carcinoma that Relapsed after Standard Surgical Excision: An Experience Report from Two Centers

Georgios Gaitanis [1,2,*], Athanasia Zampeta [1], Panagiota Tsintzou [1], Grigorios Fillis [2], Konstantinos Seretis [3], Laurence Feldmeyer [4] and Ioannis Bassukas [1]

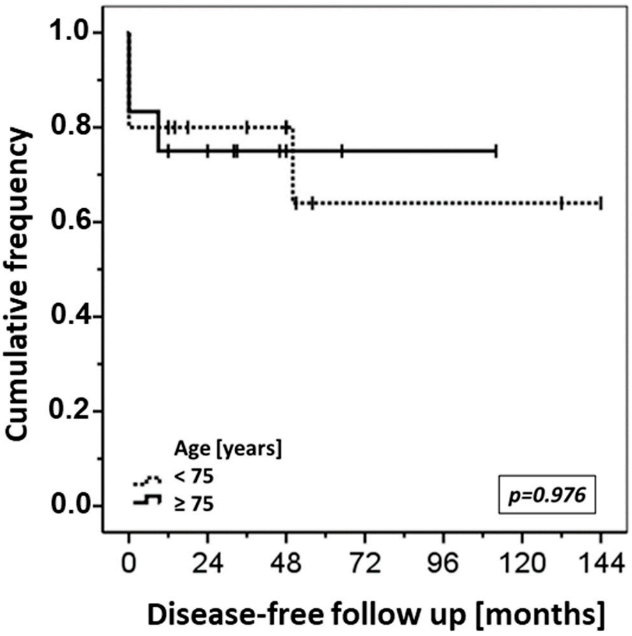

Figure S1. Immunocryosurgery for BCC relapses after surgery: Disease free tumor sites for patients older 75 years vs. younger. *p*: log rank (Mantel-Cox test).

Table S1. Patient and tumor characteristics included in the study.

	Patient			Basal cell carcinomas							
Patient/ Tumor *	Gender/Age [years]&	Co-morbidities under treatment	Number pharmaco-logical ingredients	Localization	Size (mm)	Years after last sur-gery/ Number of Sur-geries	Risk factors for re-lapse †	Histological subtype	Immuno-cryosurgery cycles	Outcome $	Follow-up (months)
1	F/76	NE	3	Cheek	23	10/1	1	Nodular	1	Clearance	46
2	F/71	CV/OR	3	Nose	16	8/1	2	Nodular	1	Clearance	48
3	F/86	CV/OP	4	Cheek	8	7/1	0	Keratotic	1	Clearance	48
4	M/45	CV/ME	3	Temple	30	2/1	2	Nodular	1	Clearance	144
5	M/71	PU/ME/CV	5	Nose	20	1/1	2	Nodular	1	Clearance	132
6	M/70	CV	N/A	Cheek	12	3/1	0	Nodular	1	Clearance	14
7	M/62	UR	0	Ear	20	1/2	1	B/S	3	Partial re-sponse	6
8 *	F/83	PU/CV	3	Periocular	8	1/1	1	Nodular	2	Clearance	24
9 *	M/76	CV/ME/UR	5	Periocular	6	1/1	1	B/S	1	Clearance	65

10 *	F/52	NE	1	Periocular	11	1/1	2	B/S	2	Clearance	28
11	M/82	PU/CV/CA/OP	5	Temple	60	4/2	3	Nodular	3	Partial response [a]	67
12	M/86	CV	4	Nose	10	3/2	1	Nodular	5	Partial response	44
13	F/80	CV/DM/ME	4	Nose	20	10/1	2	B/S	2	Clearance	32
14	M/67	CV/ME	3	Nose	15	1/1	2	Mixed (B/S, micronodular, keratotic)	1	Clearance	51
15	M/71	CV/PU/EN/NE	7	Nose	5	1/1	1	Adenoid	2	Partial response [b]	3
16	M/78	CV/PU/ME	7	Nose	5	4/1	1	Nodular	1	Clearance	112
17	M/53	none	0	Nose	15	2/1	2	Nodular	1	Partial response [b]	50
18	F/68	DM	1	Nose	10	4/1	1	Nodular	4	Partial response [b]	16
19	M/83	CV/PU/DM	3	Nose	12	4/1	2	Nodular	1	Partial response [b]	9
20	M/56	GI	0	Neck	4	10/1	0	Nodular	1	Clearance	36
21	M/78	CV/NE	5	Nose	10	3/1	1	Nodular	1	Clearance	33
22	M/81	NE	3	Nose	5	1/1	1	Nodular	1	Clearance	12
23	F/67	CV/ME	3	Nose	13	1/1	2	Nodular	1	Clearance	48
24	M/67	N/A	N/A	Temple	18	8/1	1	Nodular	1	Clearance	18
25	M/61	N/A	N/A	Nose	10	<1/1	2	Nodular	1	Clearance	12
26	M/58	N/A	N/A	Ear	6	<1/1	1	Nodular	1	Clearance	12
27	F/79	N/A	N/A	Nose	7	<1/1	1	Nodular	1	Clearance	12

*cases published previously; [&]Abbreviations. F: female; M: male; N/A: not available; NE: neurologic; CV: cardiovascular; OR: orthopedic; OP: ophthalmologic; ME: metabolic; PU: pulmonological; UR: urologic; CA: oncologic; EN: ENL; DM: diabetes melitus; GI: gastrointestinal; B/S: Basosquamous; [$] Treatment: a: vismodegib; b: surgery; [†] Risk factors for relapse [1] beyond 'relapse after surgery': H-zone area localization, size>20mm in general, size>10mm in high risk zone localization, immunosupression

References

1. Mosterd, K.; Krekels, G.A.; Nieman, F.H.; Ostertag, J.U.; Essers, B.A.B; Dirksen, C.D.; Steijlen, P.M.; Vermeulen, A.; Neumann, H.; Kelleners-Smeets, N.W.J. Surgical excision versus Mohs' micrographic surgery for primary and recurrent basal-cell carcinoma of the face: a prospective randomised controlled trial with 5-years' follow-up. *Lancet Oncol.* **2008**, *9*,1149–56.

Table S2. Immunocryosurgery for basal cell carcinoma relapses after surgery. Multivariate comparison of predictors of the treatment outcome: Cox proportional hazards model for the prediction of a disease-free tumor site ('clearance') at last follow up.

Predictor	Significance
Sex	0.371
Age [years]	0.174
Treatment for comorbidities [number of active pharmacologic ingredients]	0.831
Maximal tumor diameter [mm]	0.845
Tumor growth rate [tumor size / time since last surgery in mm/year]	0.092
Number of tumor relapses before immunocryosurgery	0.012
Risk factors for relapse before tumor relapse	0.297
